# Neonicotinoids can cause arrested pupal ecdysis in Lepidoptera

**DOI:** 10.1038/s41598-021-95284-0

**Published:** 2021-08-04

**Authors:** Niranjana Krishnan, Russell A. Jurenka, Steven P. Bradbury

**Affiliations:** 1 Department of Entomology, Iowa State University, Ames, IA USA; 2 Toxicology Program, Iowa State University, Ames, IA USA; 3 Department of Natural Resource Ecology and Management, Iowa State University, Ames, IA USA

**Keywords:** Chemical biology, Developmental biology

## Abstract

Recently, we reported a novel mode of action in monarch butterfly (*Danaus plexippus*) larvae exposed to neonicotinoid insecticides: arrest in pupal ecdysis following successful larval ecdysis. In this paper, we explore arrested pupal ecdysis in greater detail and propose adverse outcome pathways to explain how neonicotinoids cause this effect. Using imidacloprid as a model compound, we determined that final-instar monarchs, corn earworms (*Helicoverpa zea*), and wax moths (*Galleria mellonella*) showed high susceptibility to arrested pupal ecdysis while painted ladies (*Vanessa cardui*) and red admirals (*Vanessa atalanta*) showed low susceptibility. Fall armyworms (*Spodoptera frugiperda*) and European corn borers (*Ostrinia nubilalis*) were recalcitrant. All larvae with arrested ecdysis developed pupal cuticle, but with incomplete shedding of larval cuticle and unexpanded pupal appendages; corn earworm larvae successfully developed into adults with unexpanded appendages. Delayed initiation of pupal ecdysis was also observed with treated larvae. Imidacloprid exposure was required at least 26 h prior to pupal ecdysis to disrupt the molt. These observations suggest neonicotinoids may disrupt the function of crustacean cardioactive peptide (CCAP) neurons, either by directly acting on their nicotinic acetylcholine receptors or by acting on receptors of inhibitory neurons that regulate CCAP activity.

## Introduction

Neonicotinoids are among the most widely used insecticides in the world. In the United States, nearly all corn hectares and the majority of soybean and cotton hectares are planted with neonicotinoid-treated seeds, which accounts for over 80% of their total use^1^. In addition, nearly 680,000 kg of imidacloprid, thiamethoxam, and clothianidin, the three most commonly used neonicotinoids, are applied as spray, soil drench, or injection (soil/tree) formulations in agricultural land, non-crop land, and urban areas^2,3^. Not surprisingly, studies have reported potential neonicotinoid exposure to target and non-target insect species, including Lepidoptera larvae^4–8^.

In insects, neonicotinoids exert their neurotoxic effects by binding to the α4β2 subunits of the nicotinic acetylcholine receptors (nAChR) in the central nervous system. At toxic doses, receptor binding results in neuronal overstimulation, paralysis, and death^9^. Adverse outcome pathways (AOPs), which are conceptual frameworks that link a molecular initiating event to an adverse outcome^10^, have been proposed for bees at sublethal doses. In the honey bee, *Apis mellifera*, LaLone et al.^11^ proposed that nAChRs could be desensitized if exposed to prolonged but relatively low neonicotinoid doses. In addition, sublethal exposures are proposed to cause mitochondrial dysfunction, leading to alteration of Ca^2+^-calmodulin activated signal transduction. Altered Ca^2+^ transduction could, in turn, prevent translation of proteins involved in long-term memory and thereby cause abnormal foraging behavior that eventually leads to colony failure^11^. Camp and Lehmann^12^ proposed that neonicotinoids could exert similar cellular effects in bumble bees (*Bombus terrestris* and *Bombus impatiens*).

In the present paper, we report a novel effect of neonicotinoids on the development of butterflies and moths and propose associated AOPs. We characterize a unique adverse outcome, termed arrested pupal ecdysis (AE), which was first reported following neonicotinoid exposure to final instars of the monarch butterfly (*Danaus plexippus*; Nymphalidae). In the monarch, AE is characterized by a failure to complete the pupal ecdysis process; the pre-ecdysis and initial ecdysis behaviors initiated by ecdysis triggering hormone (ETH) and eclosion hormone (EH)^13^ are not affected^14^. The final instar that exhibits AE begin shedding their old larval cuticle and trachea from the abdomen but are unable to shed the larval cuticle from the head and ventral thorax. The appendages (antennae, proboscis, wings, and legs) fail to expand into the normal position of an obtect pupae. Larvae died during pupation following topical and dietary exposure to imidacloprid, clothianidin, and thiamethoxam without prior signs of intoxication^14,15^. This disruption in the pupal molt occurred at environmentally relevant doses 10 to 100-fold lower than doses that caused larval mortality due to neuronal overstimulation and paralysis^14,15^. Larval-to-larval molts were not disrupted at doses that caused AE.

These observations suggest that sublethal neonicotinoid doses (i.e., doses not causing CNS overstimulation) are disrupting neuroendocrine signaling during pupal ecdysis through a novel toxicity pathway. The roles of neuroendocrine hormones in regulation of pupal ecdysis have been studied in detail. The roles of specific neurotransmitters, however, have not been addressed to date, although several research groups note the need for further investigation^13,16^. Elucidation of neonicotinoid-induced AE symptomology in multiple Lepidoptera and development of hypothesis-based AOPs provides a framework to design future experiments to improve understanding of acetylcholine signaling in regulation of pupal ecdysis. In addition, further characterization of AE symptomology can support more informed neonicotinoid risk assessments for non-target lepidopteran species and provide new insights on neonicotinoid efficacy against late instars of pest species.

To determine if monarch larvae are unique in displaying AE, we treated six other lepidopteran larvae, belonging to Nymphalidae, Noctuidae, Crambidae, and Pyralidae, with imidacloprid. As not all species were susceptible to AE, we subsequently analyzed concentrations of imidacloprid and its metabolites in an AE-sensitive and an AE-insensitive species over time to determine if the latter metabolized and excreted larger quantities of imidacloprid prior to pupal ecdysis. Results from this experiment also shed light on the range of internal imidacloprid doses needed to elicit AE. By treating larvae with imidacloprid at time points before and after head capsule slippage (HCS), we identified when final instars are susceptible to AE. Finally, to document the phenotypic events resulting in AE, the pupal ecdysis process was documented in fine temporal detail with control and imidacloprid-treated larvae. To formulate proposed AOPs for imidacloprid-induced AE, results from this series of experiments were interpreted in light of literature addressing neuroendocrine control of pupal ecdysis.

## Results

### Species’ susceptibility to AE

**Figure 1:**
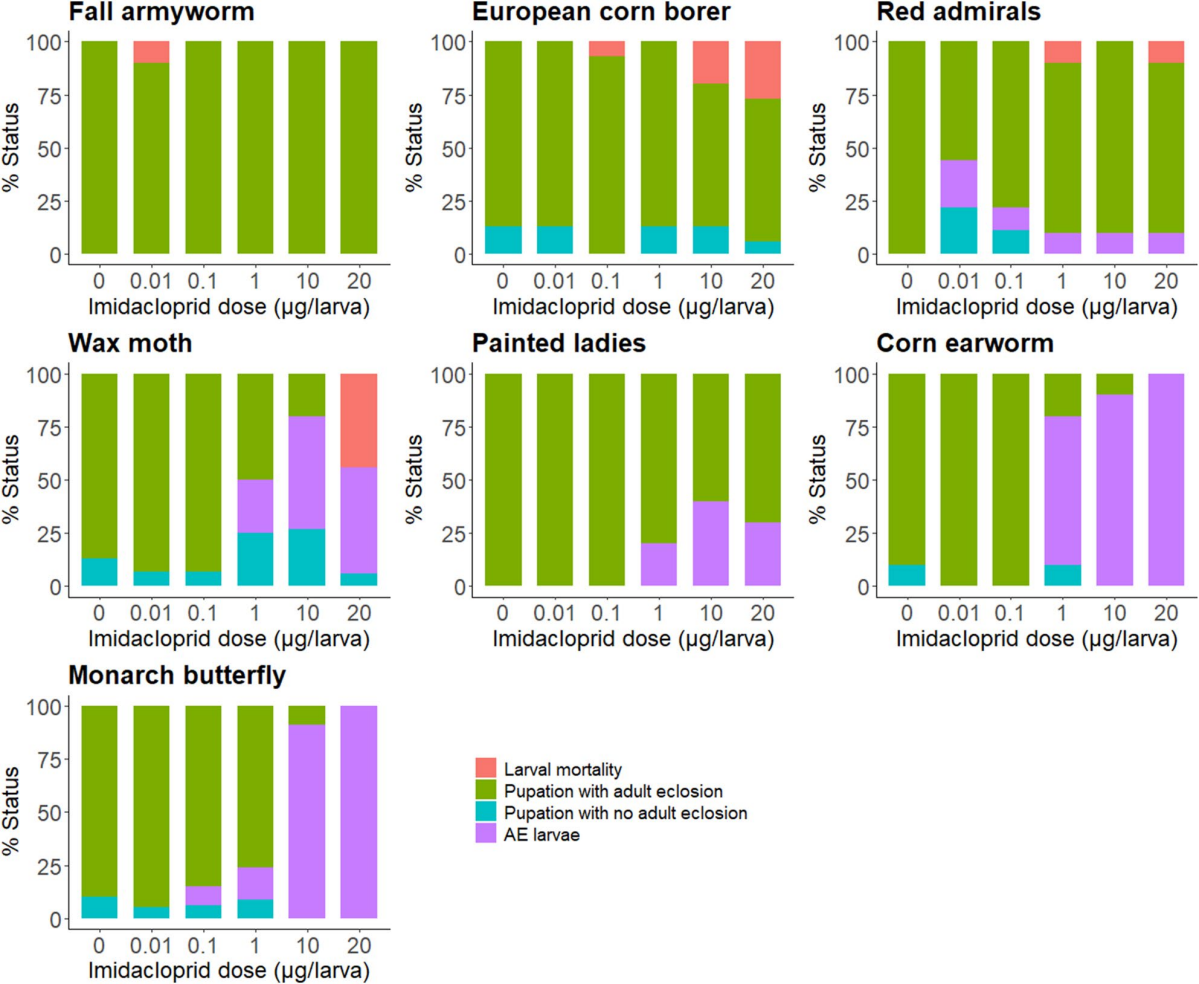
Stacked barplots depicting percent of imidacloprid-treated final instars that had larval mortality, pupated with adult eclosion, pupated without adult eclosion, and arrested pupal ecdysis (AE). Number of larvae treated per dose: 10 for fall armyworms, red admirals, painted ladies, and corn earworms, 15 for European corn borers and wax moths, and for monarch butterflies 22–33 at 0–10 µg doses and 10 at the 20 µg dose.

**Figure 2:**
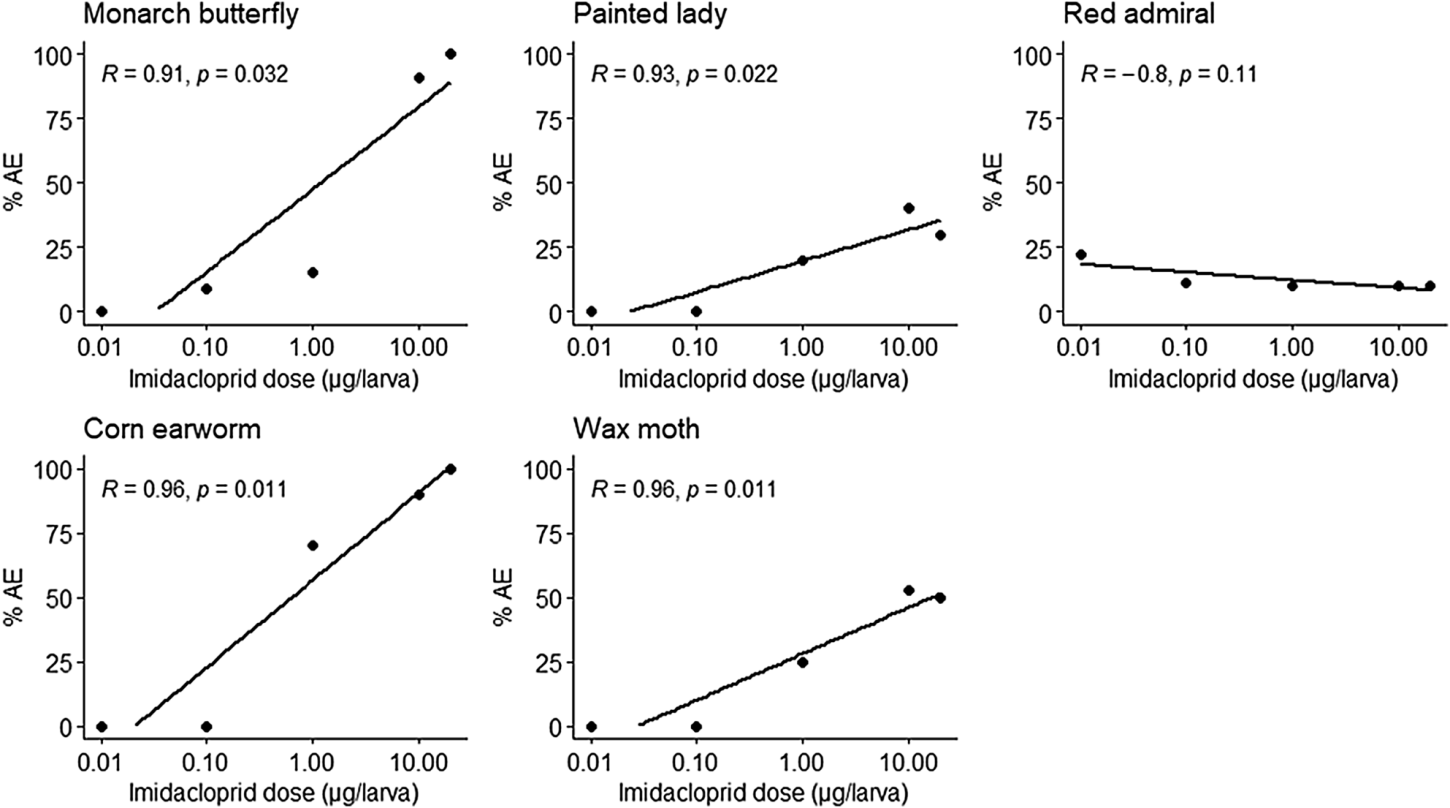
Correlation between imidacloprid dose (µg/larva) and percent arrested pupal ecdysis (AE) in monarch butterfly (n = 152), painted lady (n = 60), red admiral (n = 60), corn earworm (n = 60), and wax moth (n = 92) final instars. Pearson’s correlation coefficient was used to calculate R and significance value for each species. Note that the x-axis is on a logarithmic scale.

**Figure 3:**
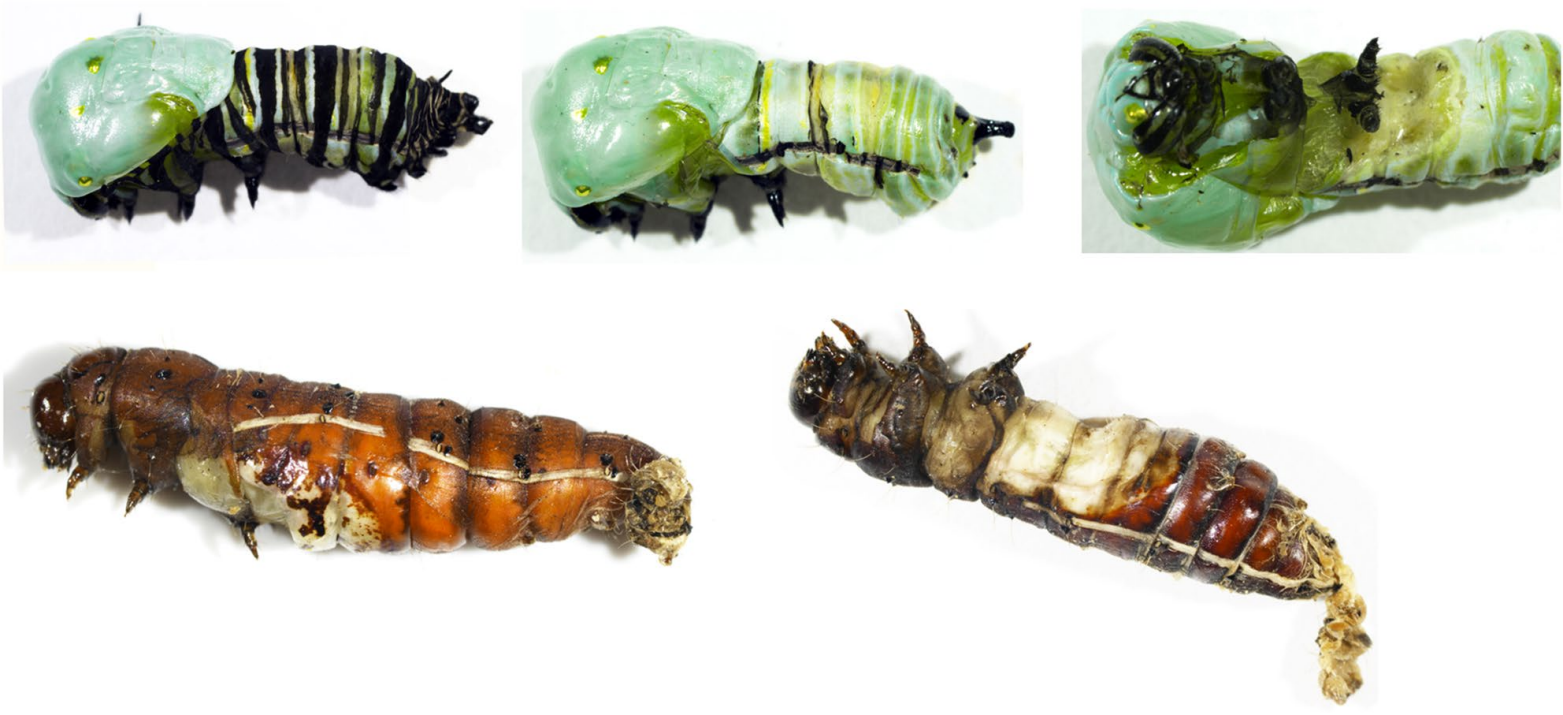
Arrested pupal ecdysis in representative fifth instar monarch butterfly (top row) and sixth instar corn earworm (bottom row) treated with 20 µg imidacloprid. Careful removal of old larval cuticle from monarchs (top row middle and right) and an examination of corn earworms showed complete pupal cuticle on the dorsal and posterior side and partially shed tracheal cuticle. In both species, appendages had not expanded and the ventral side of the first abdominal segments were not sclerotized.

AE was observed in monarchs, corn earworms, wax moths, painted ladies, and red admirals that were topically treated with 1 to 20 µg imidacloprid (Fig. 1). Fall armyworms and European corn borers were recalcitrant to AE even following a 100 µg imidacloprid treatment (Table S1). A positive correlation (*p* ≤ 0.032) between dose and percent AE was seen in all susceptible species except red admirals (Fig. 2). Removal of larval cuticle from AE butterfly larvae and a closer examination of AE moth larvae showed incomplete shedding of larval trachea, a partially shed larval cuticle at the posterior end of the abdomen, a partially complete pupal case (partially complete in that larval cuticle of the head and legs could not be removed to determine if there was an underlying pupal cuticle), and untanned cuticle on the ventral side where the pupal wing cuticle would normally expand (Figs. 3 and S1).

**Figure 4:**
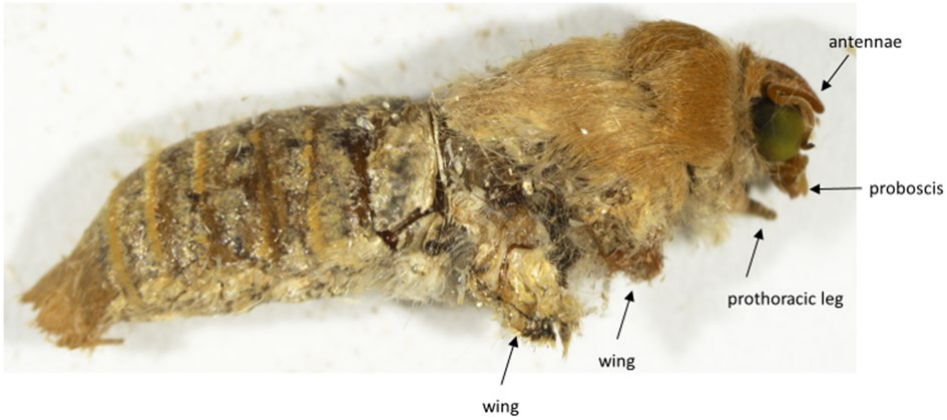
Corn earworm larvae that exhibited arrested pupal ecdysis were allowed to develop as adults. Careful removal of larval and pupal cuticle after adult development shows an adult with appendages (labeled) that did not expand.

In monarchs, AE was observed in 100% of larvae that did not undergo complete pupation at doses ≤ 20 µg (Fig. 1). Over 90% of larvae exposed to 10 and 20 µg imidacloprid exhibited AE. At higher doses, mortality mostly occurred through imidacloprid’s primary mode of action (i.e., overstimulation of the CNS) (Table S1), which included cessation of feeding, paralysis, and bleeding symptoms. In painted ladies and red admirals, 50 to 100% of treated larvae that failed to pupate exhibited AE; however, the percent AE at any dose did not exceed 40% (Fig. 1). For all three butterfly species, larvae expressing AE either died following emergence of the pupal cuticle on the thorax or at the “J” stage, often accompanied by external bleeding. The moth species that expressed AE had a visible pupal cuticle on the abdomen and the dorsal and posterior sides of the thorax. In wax moths, all larvae that failed to pupate at doses up to 10 µg imidacloprid had AE (53% of treated larvae), while larvae treated with the 20 µg dose either exhibited AE or died through imidacloprid’s primary mode of action. Four of the 10 AE larvae we dissected initiated, but did not complete, adult development. In corn earworms, all treated larvae that did not successfully pupate had AE, with the 10 and 20 µg imidacloprid doses producing AE in 90 and 100% of larvae, respectively (Fig. 1). Of note, AE often did not lead to immediate mortality in corn earworms. Instead, adults developed within the unshed larval and pupal cuticles. Removal of the old cuticles approximately two weeks after treatment revealed a completely formed adult except the appendages (proboscis, antennae, wings, and legs) were not expanded (Fig. 4).

Based on observations taken every 24 h, there were no differences between control and treated larvae in the number of days it took to initiate pupal ecdysis following treatment (*p* > 0.27 for all species; see Table S2). In all species, doses that produced at least two pupae had adult eclosion rates ranging from 61 to 100% (Fig. 1).

### Imidacloprid toxicokinetics in AE-susceptible and unsusceptible species

**Figure 5:**
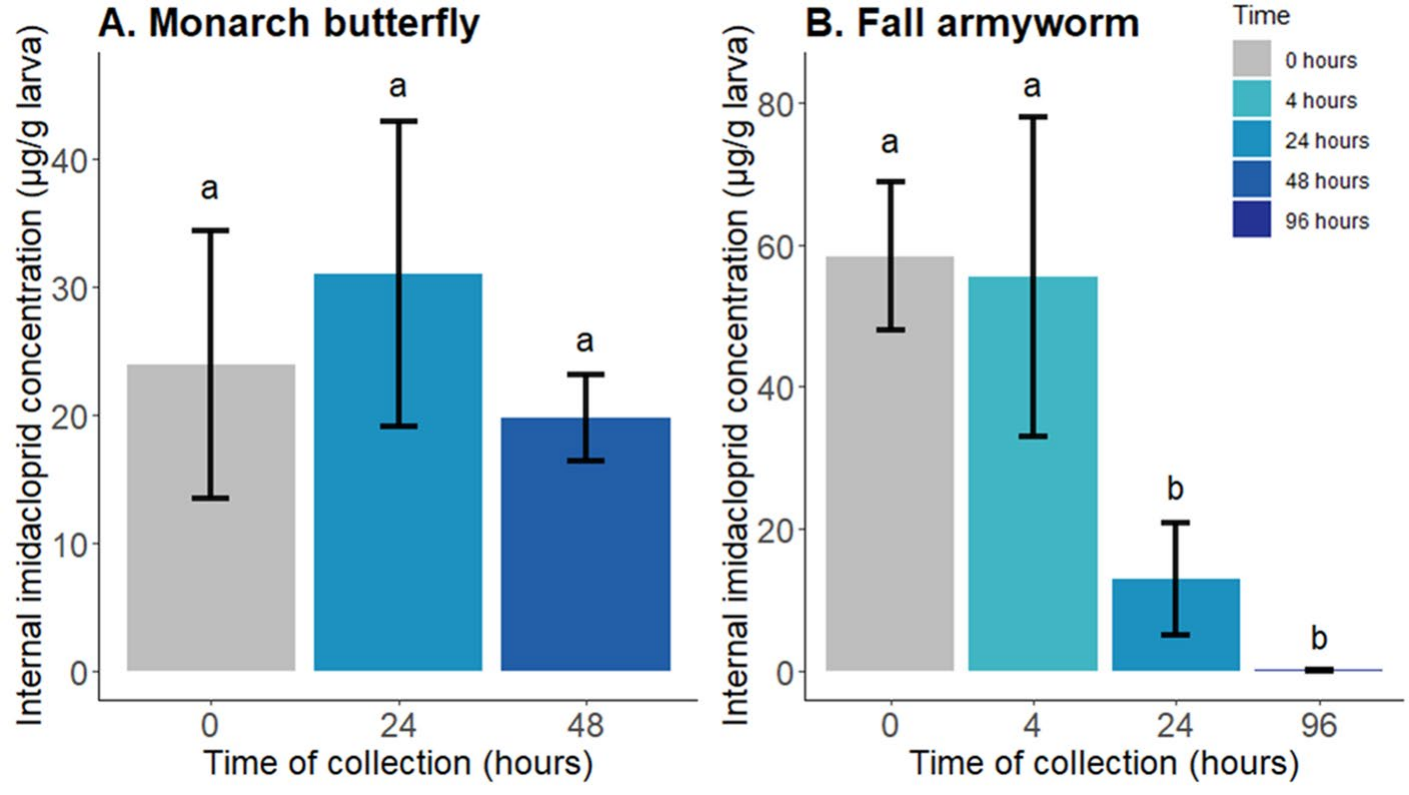
Mean internal concentrations (± standard deviation) of imidacloprid in monarch butterfly (**A**) and fall armyworm (**B**) final instars. Larvae were collected at the following time points after topical treatment with 20 µg imidacloprid : 0 h (both species; larvae), 4 h (fall armyworm; larvae), 24 h (both species; larvae), 48 h (monarch butterfly in arrested pupal ecdysis), and 96 h (fall armyworm as pupae). For each species, different lower-case letters above bars denote significant differences (*p* < 0.05) between control and treated larvae using Dunnett’s test for multiple comparisons.

Sixth instar fall armyworm (an AE-unsusceptible species) that were topically treated with 20 µg imidacloprid and collected within five minutes of treatment contained a mean (± SD) of 59 (± 11) µg/g imidacloprid (Fig. 5); two of the four larval samples also had detectable 5-hydroxy imidacloprid below the limit of quantification (< 0.02 µg/g; Table S3). Larvae collected 4 h after treatment had a similar imidacloprid concentration (56 ± 23 µg/g) and three of the five samples had one or both imidacloprid metabolites. Larvae collected 24 h after treatment had four-fold lower imidacloprid concentrations (13 ± 7.9 µg/g) and four of the five samples contained one or both metabolites. The parent imidacloprid concentration in larvae at 24 h was significantly lower than at 0 h (t-ratio: − 5.135; *p* = 0.0003; Fig. 5). Pupation occurred approximately four days after treatment and only two of the five pupal samples had detectable concentrations of imidacloprid; in both samples the concentrations were lower than the limit of quantification. Neither of the metabolites were detected in the pupal samples.

Fifth instar monarch butterflies that were topically treated with 20 µg imidacloprid and collected within 5 min of treatment contained a mean of 24 (± 10) µg/g imidacloprid (Fig. 5). Fifth instars collected 24 h (larval stage) and 48 h (AE stage) after treatment contained similar imidacloprid concentrations (31 ± 12 and 20 ± 3.4 µg/g, respectively). The two imidacloprid metabolites were not detected in any of the monarch samples (Table S3). No differences between parent imidacloprid concentrations were found in larvae collected at the different time points post exposure (F = 1.8496; *p* = 0.1994).

### Effect of imidacloprid at various times prior to pupation

**Figure 6:**
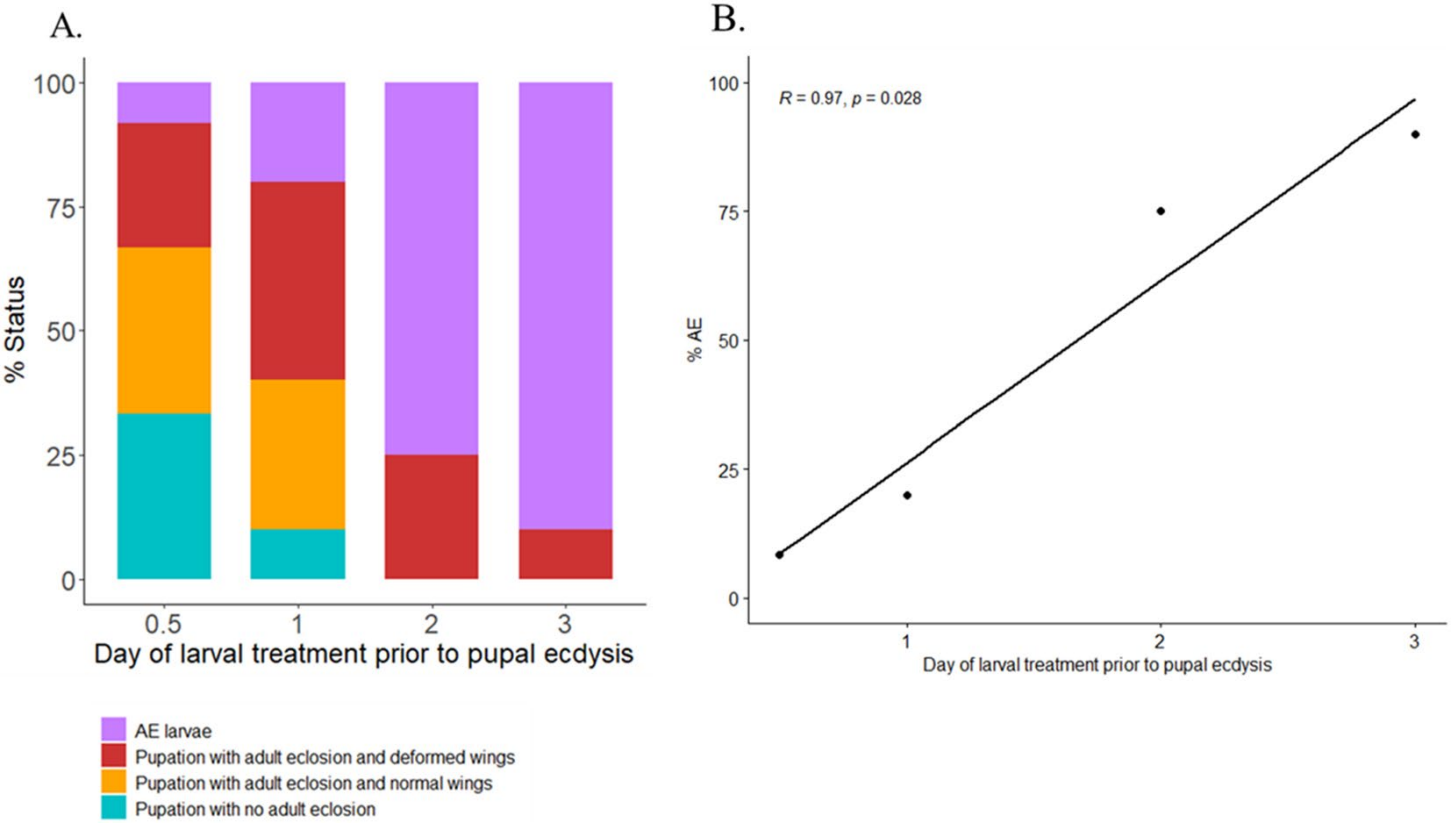
Corn earworm larvae were treated topically with 20 µg imidacloprid at four time points prior to pupal ecdysis. (**A**) Stacked barplot depicting the percent of imidacloprid-treated final instar corn earworms that had arrested pupal ecdysis (AE), pupation with no adult eclosion, pupation with adult eclosion and deformed wings, and pupation with adult eclosion and normal wings. (**B**) Correlation between day of treatment and percent AE. Pearson’s correlation coefficient was used to calculate R and significance. Eight to 12 larvae were treated per time point. All of the control larvae (n = 4 to 6 per time point) had successful pupation with normal adult eclosion.

Following topical treatment with 20 µg imidacloprid, sixth instar corn earworms treated prior to HCS (ca. 3 days before pupation) had the highest level of AE (90%) (Fig. 6A). Larvae treated just after HCS (ca. 2 days before pupation) had 75% AE. Larvae that were treated ca. 12 h (ca. 1 day before pupation) and 23 h (ca. 0.5 day before pupation) post HCS, had 20 and 8% AE, respectively, with the remaining larvae successfully undergoing pupal ecdysis. A significant correlation between day of larval treatment and percent AE was observed (*p* = 0.028; Fig. 6B). Nearly 90% of the AE larvae survived and developed to the adult stage and a third of these attempted to emerge but only their abdomen lost the pupal cuticle (data not shown). Of those larvae that successfully pupated, a small percentage had wrinkled appendages, loss of hemolymph, and bloated cuticle around the wings (Figure S2A). Of all the larvae that successfully pupated, only 23% (n = 5) emerged normally, though their eclosion was delayed by one to two days with respect to controls. The remaining pupae either did not emerge or emerged with deformed and uninflated wings (Figs. S2B and 6A).

While monarch larvae also showed high rates of AE when topical treatments were applied 2–3 days before pupation^14^, the pattern of AE expression following dietary exposure differed. Final-instar monarchs that were 2–3 days from pupation and fed 0.5 µg of imidacloprid/g leaf for 24 h, followed by 24-h feeding on untreated leaves, pupated normally (Table S4). However, final-instar monarchs that were 1–2 days from pupation and only provided leaves treated with imidacloprid for 24 h expressed AE. These differences in AE expression based on topical and dietary exposure routes and timing were also observed in previous work^14,15^. To help explain these differences, imidacloprid concentrations within monarch samples were measured. Pupal samples from larvae that fed on treated leaves for 24 h followed by feeding on untreated leaves for 24 h had imidacloprid concentrations below the level of quantification (> 0.02 ug/g). Larvae with AE that were not provided untreated leaves had a mean (± SD) of 0.14 (± 0.06) µg/g imidacloprid (Table S4). These results suggest differences in AE susceptibility between topical and dietary exposure may be attributed to faster rates of imidacloprid excretion following dietary exposure.

### Observational details on pupation in corn earworms

On average, both acetone-treated and imidacloprid-treated sixth instar corn earworms initiated HCS 42 h after exposure. Pupal ecdysis was initiated at a mean (± SD) of 68 (± 3.7) and 73 (± 7.5) hours post exposure, respectively (Table 1). Following HCS, the imidacloprid-treated larvae initiated the ecdysis motor process approximately 5 h later than the controls, i.e., 31 (± 6.4) versus 26 (± 1.7) hours for controls. This difference was statistically significant at α = 0.05 level (t = − 2.859; *p* = 0.0139).

**Table 1:** Time to head capsule slippage and pupation or arrested pupal ecdysis for corn earworm sixth instars topically treated with acetone or 20 µg imidacloprid^a^.

Treatment (n)	Percentage	Mean (± SD) hours to start of HCS [A]^b^	Mean (± SD) hours to start of pupation/AE [B]^b^	Mean (± SD) hours from start of HCS to start of pupation/AE [B-A]
Acetone (n = 12 larvae)	Pupation: 100%	42 (± 3.7) (range: 39 to 49 h)	68 (± 3.7) (range: 64 to 75 h)	26 (± 1.7) (range: 22 to 29 h)
Imidacloprid (n = 12 larvae)	AE: 100%	42 (± 3.4) (range: 39 to 49 h)	73 (± 7.5) (range: 68 to 94 h)	31 (± 6.4) (range: 26 to 49 h)

*HCS* head capsule slippage, *AE* arrested (pupal) ecdysis, *SD* standard deviation.

^a^Larvae were approximately 2-day old sixth instars at the time of treatment.

^b^Hours to HCS and pupation/AE were calculated from time of treatment.

Acetone-treated corn earworm larvae took ca. 10 min to complete pupal ecdysis, from initiation of tracheal shedding through complete shedding of larval cuticle. After ca. 1 to 2 min following initiation of the posterior abdomen larval cuticle and tracheal shedding, the larval cuticle at the dorsal thorax split along the ecdysial line and the pupal cuticle started to emerge. Approximately one minute later, the larvae turned over, exposing the ventral side and began shedding its larval cuticle from the anterior. Expansion of the pupal appendages began just before ecdysis and continued during and after ecdysis. Immediately following ecdysis, the wing length was ca. 5 mm and expanded to 10 mm over the next 15 min prior to cuticle hardening and tanning.

Imidacloprid-treated corn earworm larvae behaved as previously described; they initiated, but did not complete, abdominal cuticle and tracheal shedding. While the larval cuticle at the dorsal thorax did not split in most larvae, some did split the dorsal thoracic cuticle along ecdysial lines ca. 5 min after initiation of abdominal cuticle and tracheal shedding. None of the imidacloprid-treated larvae progressed beyond this point, i.e., they did not turnover to expose the ventral side and begin shedding their larval cuticle. These larvae also did not expand their appendages. For two AE corn earworm larvae that continued the ecdysis process past the abdominal cuticle and tracheal shedding, the larval cuticle was removed from the abdomen but the thoracic and head larval cuticle remained. After approximately 30 min, we carefully removed the larval cuticle from the thorax and head. The larval cuticle over the legs and head could be successfully pulled off and revealed a complete pupal cuticle except the appendages were not expanded (Figure S3).

## Discussion

In the following sections, we interpret and integrate results from our experiments to gain a better understanding of AE symptomology and differences in interspecies susceptibility. Based on our observations and review of the insect ecdysis literature, we propose two AOPs for neonicotinoid-induced AE. We do note AE-like effects were reported by Bargar et al.^17^ when they chronically exposed monarch larvae to clothianidin-treated milkweed plants, and by Heneberg et al.^18^ when they treated crabronid wasp prepupae with neonicotinoids. While these studies are broadly concordant with our observations, they do not provide detailed descriptions of the symptoms or their time course. There are currently no published papers that attempt to elucidate the potential mechanism(s) by which sublethal doses of neonicotinoid insecticides could cause AE.

### Conservation of neonicotinoid-induced AE across Lepidoptera

High rates (50 to 100%) of AE were observed in monarchs (Nymphalidae), corn earworms (Noctuidae), and wax moths (Pyralidae). Painted ladies and red admirals (both Nymphalidae) had lower rates (10 to 40%) of AE. The larval cuticle of these two species are densely covered with hairs and it is possible imidacloprid was not completely absorbed. However, even with dietary exposures, painted ladies are approximately 70-fold less sensitive to the neonicotinoid clothianidin when compared to monarchs^7,15^ suggesting additional toxicokinetic or toxicodynamic factors contribute to painted ladies relatively low sensitivity to neonicotinoids. European corn borers (Crambidae) and fall armyworms (Noctuidae) were recalcitrant to AE. These findings suggest susceptibility to AE, and corresponding low toxicity of imidacloprid, may not be related to lepidopteran phylogenetic similarity. As both non-target (butterfly) and pest (moth) species show susceptibility to AE, these findings have implications in lepidopteran non-target conservation in agricultural landscapes and in lepidopteran pest management.

To assess if interspecies variability in AE sensitivity could be due, in part, to differences in toxicokinetics, we topically treated monarch and fall armyworm larvae and quantified internal concentrations of imidacloprid and two of its toxic metabolites. In monarchs, internal imidacloprid concentrations remained stable over 48 h and neither of the metabolites were detected in any samples. In fall armyworms, the internal imidacloprid concentration fell rapidly; 24-h larval samples had four-fold lower imidacloprid concentrations than the 0-h samples and, by 96-h, imidacloprid was undetectable. Additionally, imidacloprid metabolites were detected at the 0, 4, and 24-h sampling points. While these data show fall armyworms metabolize and excrete imidacloprid more efficiently than monarchs, examination of insecticide uptake and distribution in the CNS and endocrine-active tissues over time is needed to fully assess the extent of toxicokinetic differences between the species. We also cannot rule out toxicodynamic considerations that could explain the differences in species susceptibility, e.g., interspecies differences in imidacloprid binding potential to nAChRs.

### Rationale for a novel mode of action

There are several observations that suggest imidacloprid-induced AE occurs through a novel toxicity pathway. When imidacloprid acts through its primary mode of action, larvae show signs of CNS poisoning (cessation of feeding, paralysis, bleeding) within 24 h and die soon thereafter^14^. Early instars are more sensitive to the primary mode of action^14^, possibly due to their smaller body weights. In contrast, with AE we observe no neurotoxic symptoms and instead observe delayed mortality, greater sensitivity of final instars, and a narrow temporal window of susceptibility at doses below the threshold that elicit symptoms associated with the CNS intoxication.

#### Delayed mortality with no neurotoxic symptoms

Monarchs, corn earworms, painted ladies, and red admirals that exhibited AE at doses ≤ 20 µg and wax moths that exhibited AE at doses ≤ 10 µg showed no detectable symptoms prior to ecdysis. Most AE larvae had initiated pupal ecdysis 2 to 6 days after treatment, similar to controls. Fifth-instar corn earworms topically treated with 20 µg imidacloprid molted to the sixth instar with no symptoms and then exhibited AE (Figure S4) 6 to 8 days after treatment. Additionally, corn earworm larvae that exhibited AE did not die immediately and developed adult features.

#### Greater sensitivity of final instars

Previously we had published dietary toxicity data for monarch second, third, and early fifth instars provided imidacloprid-treated milkweed leaves for 24 to 48 h^14,15^. A dietary exposure equivalent to 1.5 to 7.0 µg of imidacloprid/g larva caused 0 to 5% mortality, with none attributed to AE. However, when late fifth instars were provided an imidacloprid dose of 0.76 µg/g, 100% mortality occurred, all through AE^15^. With topical exposures, a 9.1 µg/g imidacloprid dose caused 90% mortality in fifth instars; doses that were two (17 µg/g) and six (56 µg/g) times greater were needed to cause similar levels of mortality through CNS intoxication in first and third instar larvae, respectively^14^.

#### AE effects at low doses

In monarch fifth instars, the average doses that caused over 90% mortality through AE ranged from 0.76 (dietary exposure) to 9.1 (topical exposure) µg/g larvae; doses of 124 (dietary exposure) and 91 (topical exposure) µg/g are needed to cause similar rates of mortality through imidacloprid’s primary mode of action^14,15^. These results indicate that the imidacloprid doses that cause AE in monarchs are 10 (topical exposure) to 160 (dietary exposure) fold lower than doses that cause larval mortality through the primary neurotoxic mode of action.

#### Narrow temporal window of susceptibility

AE symptomology is specific to larval to pupal ecdysis; in both monarchs and corn earworms, larval to larval ecdysis was unaffected^14,15^ (Figure S4). Further, in sixth instar corn earworm larvae, we observed high rates of AE (75 to 100%) when topical imidacloprid exposures occurred ca. 1 day before HCS or immediately after HCS. If larvae were treated 0.5 or 1 day after HCS, the vast majority of larvae (80 to 92%) formed complete pupae (Fig. 6). Of note, HCS occurs after decline of the second ecdysteroid peak, which leads to the release of ETH peptides that initiate the ecdysis process^13^. While additional toxicokinetic data that provide a more refined time course of insecticide absorption and distribution are needed, the findings to date nevertheless suggest that neonicotinoids disrupt pupal ecdysis within a specific and relatively small window of time.

### Interpretation of AE symptomology

As described previously, AE typically does not lead to immediate mortality in corn earworms; consequently, an analysis of these larvae provided useful insights on the time course of key events. We have observed the following:

1. AE occurred after initiation of pupal ecdysis and was characterized by incomplete shedding of trachea and larval cuticle.
2. AE larvae could form complete pupae and adults (i.e., development was not affected); however, the pupal and adult appendages were unexpanded/uninflated.
3. High rates of AE were observed if topical imidacloprid exposure occurred just prior to pupal HCS.
4. Imidacloprid did not disrupt larval to larval ecdysis.
5. Imidacloprid delayed initiation of pupal ecdysis.
6. Imidacloprid-treated larvae that successfully pupated had delayed adult eclosion.

Based on these observations, we propose neonicotinoids are interfering with the function of crustacean cardioactive peptide (CCAP) neurons. Neurons containing CCAP help maintain ecdysis, which is initiated by ETH and EH. In fruit flies (*Drosophila melanogaster*), CCAP neurons are necessary for pupal leg and wing expansion, timely eclosion, and adult wing inflation^19,20^. In the red flour beetle, *Tribolium castaneum*, knockdown of the gene encoding CCAP resulted in arrested adult ecdysis, but the pupae had normal pre-ecdysis behaviors^21^. CCAP neurons are also needed for successful pupal, but not larval, ecdysis. Failed pupal ecdysis following ablation of CCAP neurons is characterized by incomplete shedding of larval trachea and failure to extend appendages^19,22^. In the hemimetabolous insect *Rhodnius prolixus*, knockdown of CCAP has also been reported to delay initiation of the ecdysis motor process^23^.

Different subsets of CCAP neurons release CCAP, myoinhibitory peptides (MIPs), and bursicon peptides during and after ecdysis^13^. Bursicon is responsible for cuticle sclerotization and tanning; however, the pupal and adult cuticle of neonicotinoid-treated corn earworm larvae have coloration and hardness similar to control cuticle (the unsclerotized cuticle on the ventral side of AE pupae can be attributed to unexpanded appendages). In fruit flies, bursicon subunits are also believed to be necessary for successful pupal ecdysis and adult wing inflation^22^. Of note, just prior to pupal ecdysis in fruit flies, there is an emergence of 12 ‘late’ CCAP neurons that differentiate to express CCAP and bursicon. These neurons alone are sufficient to initiate pupal ecdysis^20^. These ‘late’ CCAP neurons are necessary for pupal leg extension, while the ‘early’ CCAP neurons are necessary for adult wing inflation. In total, these observations in fruit flies suggest that in Lepidoptera, the neonicotinoids may be disrupting signaling from both ‘early’ and ‘late’ CCAP neurons, including subsets of bursicon-expressing CCAP neurons. Although the CCAP neurons are conserved in arthropods^24^, including Lepidoptera^25^, it is not known if the ‘early’ and ‘late’ CCAP neuron functionality is conserved.

### Proposed adverse outcome pathways for AE in Lepidopteran

**Figure 7:**
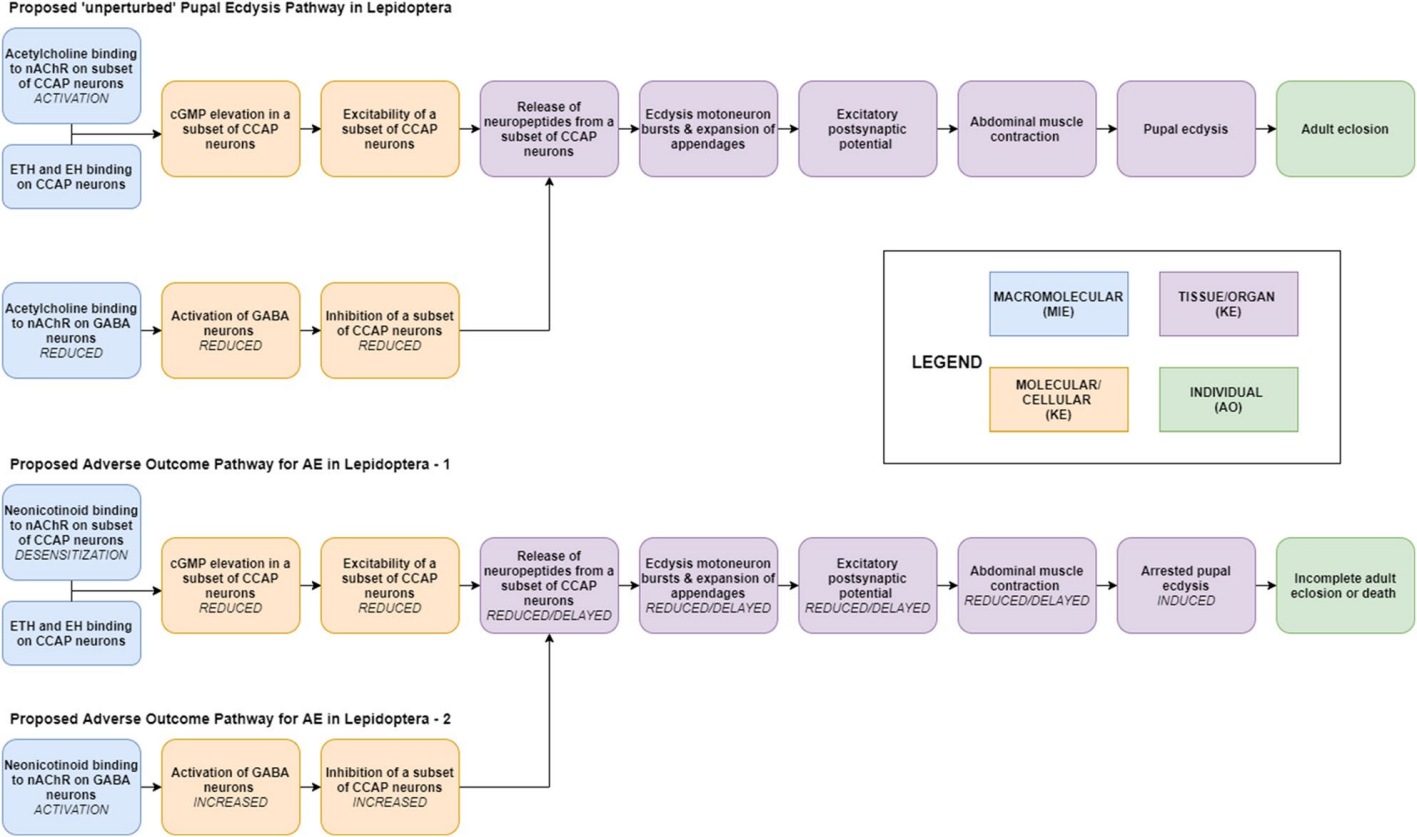
Proposed adverse outcome pathways that elucidate how neonicotinoid treatment could lead to arrested pupal ecdysis (AE). *MIE* molecular initiating event, *KE* key event, *AO* adverse outcome.

We propose two AOPs that provide testable hypotheses to elucidate the molecular initiating events and resultant key events associated with neonicotinoid-induced AE (Fig. 7). In developing the proposed AOPs, we reviewed AOPs for neonicotinoid-induced effects on honeybee colony death^11^ and an AOP for ecdysone agonists that leads to lethal molting disruption^26^. We also reviewed the literature addressing neuroendocrine pathways that initiate and regulate ecdysis.

#### Molecular initiating event

Consistent with their primary CNS-based mechanism of action, we propose that AE is also initiated by neonicotinoid binding to nAChRs, but in a distinct group of neurons. While the neonicotinoid’s primary mode of action involves their agonism of acetylcholine at nAChRs, it is possible AE could be due to interactions at other receptor binding sites. However, we previously reported increased rates of AE in monarch fifth instars topically treated with chlorpyrifos^14^, which is an acetylcholine esterase inhibitor that has the net effect of increasing acetylcholine signaling. To determine if this response could be replicated in another susceptible species, we treated sixth instar corn earworm larvae with chlorpyrifos. Of the ten corn earworms topically treated with 140 µg chlorpyrifos, seven exhibited AE (data not shown). These findings suggest that disruption of acetylcholine signaling pathways may be central to AE.

In holometabolous insects, several steps occur sequentially to initiate pupal ecdysis. In moths, following the release of ETH, pre-ETH (PETH), and ETH-associated peptide (ETH-AP) from Inka cells, various neurons are activated including the L3, 4 neurons that release diuretic hormones and kinins, VM neurons that release EH, and the CCAP neurons^13,19,22^. In fruit flies*,* subsets of CCAP neurons possess different receptors including ETHR, EHR, nAChR, muscarinic acetylcholine receptors (mAChR), GABA, and glutamate receptors^16,27–29^. Perturbation of normal ETH, EH, acetylcholine, GABA, and glutamate signaling could interfere with CCAP activation and/or secretion of neuropeptides and cause symptoms similar to the neonicotinoid-induced AE. In this regard, Vömel and Wegener^29^ reported that in fruit flies, the vast majority of CCAP neurons respond to acetylcholine (which binds to nAChR and mAChR) and nicotine (which binds to nAChR). Desensitization of response was observed at higher nicotine concentrations. Subsets of CCAP neurons that were inhibited by GABA were stimulated by acetylcholine*. *In vivo studies indicated nicotine could activate presynaptic inhibitory neurons, which are likely GABA neurons.

#### Key events

Studies in tobacco hornworm (*Manduca sexta*) have shown that cGMP production can take place in certain nitric oxide (NO)-sensitive CCAP neurons only during pupal ecdysis^30^. In turn, elevated cGMP can lead to increased excitability of CCAP neurons^31^. Zayas et al.^32^ and Mannai et al.^33^ showed that nicotinic acetylcholine receptors can control cGMP levels by coupling to NO production. While cGMP elevation in CCAP neurons mark the activation of the ecdysis circuitry, release of CCAP neuropeptides and initiation of ecdysis behavior takes place 30 min later. This delay in release of peptides is likely caused by a descending inhibitory input^34,35^. In fruit flies, GABA neurons are partly responsible for this inhibition (i.e., delayed activation) of CCAP neurons^36^.

Neonicotinoids could initiate AE through two pathways (Fig. 7). They could directly bind to nAChRs on a subset of CCAP neurons and cause receptor overstimulation and desensitization^11^. Alternatively, neonicotinoids could bind to nAChRs on GABA neurons that inhibit CCAP neurons prior to ecdysis. Continued activation of GABA-releasing neurons could delay or diminish disinhibition of a subset of CCAP neurons. Both these AOPs would have a net effect of reduced cGMP elevation and reduced activation of CCAP neurons. In turn, reduced release of CCAP neuropeptides would attenuate the ecdysis motor process (weaker ecdysis movements and abdominal contractions) and prevent expansion of appendages, consistent with observed AE symptomology and mortality.

## Conclusions

We describe a unique adverse effect caused by neonicotinoid insecticides in butterflies and moths. Specifically, we show that neonicotinoids cause failure of pupal (but not larval) ecdysis at doses below those that cause mortality through CNS neurotoxicity. We propose that neonicotinoids at lower doses, which do not elicit symptomology associated with CNS neurotoxicity, interfere with the release of neuropeptides involved in the ecdysis motor program and expansion of pupal appendages. While the majority of lepidopteran species we studied were susceptible to AE, some species were recalcitrant to the effect, which may, in part, be due to species’ differences in neonicotinoid toxicokinetics. Further investigation of the mechanisms through which neonicotinoids induce AE will likely aid in better understanding of the insect molting and metamorphosis processes and help refine neonicotinoid risk assessments for non-target lepidopteran species of conservation concern. We propose two AOPs that hypothesize different molecular initiating events through which neonicotinoids could attenuate signaling from CCAP neurons that are necessary for continuation of the ecdysis motor process and extension of appendages during the larval to pupal molt.

## Materials and methods

### Insect rearing

Monarch butterfly and European corn borer (*Ostrinia nubilalis*; Crambidae) larvae were obtained from colonies established by the U.S. Department of Agriculture in Ames, IA. Monarch larvae were fed on greenhouse-grown tropical milkweed (*Asclepias curassavica*) leaves^14^, while European corn borer larvae fed on an artificial diet^37–39^. Larvae of both species were reared in incubators maintained at 26.6 °C, 65% relative humidity, and 16:8 light: dark cycle.

Corn earworm (*Helicoverpa zea*; Noctuidae) and fall armyworm (*Spodoptera frugiperda*; Noctuidae) eggs were purchased from Benzon Research, Carlisle, PA, and painted lady (*Vanessa cardui*; Nymphalidae) eggs were purchased from Carolina Biological Supply Co., Burlington, NC. Larvae were fed ad libitum on an artificial diet (Stonefly Heliothis Diet, Ward’s Science) and were reared in an incubator maintained at 26 °C and 14:10 light: dark cycle.

Ovipositing female red admiral butterflies (*Vanessa atalanta*; Nymphalidae) were collected from common nettle (*Urtica dioica*) in June 2020 from prairies in Story and Boone counties, IA, USA. Larvae that hatched from eggs laid by the captured females were fed common nettle leaves. Wax moth (*Galleria mellonella*; Pyralidae) larvae were purchased from a commercial store in Ames, IA, USA. Larvae were fed multigrain baby cereal (Gerber brand) that was mixed with water to achieve consistency of thick peanut butter. Red admirals and wax moths were reared in an incubator at 26.6 °C, 80% relative humidity, and 16:8 light: dark cycle.

### Chemicals and insecticide solutions

Analytical grade imidacloprid (IUPAC name: *N*-[1-[(6-chloropyridin-3-yl)methyl]-4,5-dihydroimidazol-2-yl]nitramide; CAS number: 138261–41-3; Percentage purity: 100%) was purchased from Sigma-Aldrich. To prepare insecticide stock solutions for topical and dietary bioassays, certified ACS reagent grade acetone, certified ACS reagent grade dimethylformamide (DMF), and Silwet L-77 were purchased from Fisher Scientific.

For the topical bioassays, 20 and 10 mg/mL imidacloprid stock solutions were prepared in acetone. Tenfold serial dilutions through 0.01 mg/mL imidacloprid were made with the 10 mg/mL solution. For the dietary bioassay, 10 mg of imidacloprid was dissolved in 10 mL of DMF; the stock solution was diluted ten-fold to 0.1 mg/mL with DMF. This concentration was serial diluted with a suspension of 0.1% silwet: water to obtain a 0.01 mg/mL (or 0.01 μg/μL) imidacloprid suspension.

### Toxicity bioassays

All bioassays were conducted from January 2020 to January 2021 at the environmental conditions specified in “Insect rearing” section.

#### Species’ susceptibility to AE

Studies were conducted on 1- or 2-day-old final-instar larvae of European corn borers (fifth instar), corn earworms (sixth instar), fall armyworms (sixth instar), painted ladies (fifth instar), red admirals (fifth instar), and wax moths (sixth instar). All larvae were placed in individual rearing containers and randomly assigned a treatment prior to starting a bioassay. One μL of acetone or an imidacloprid-acetone solution was placed on the dorsal prothorax using a 10 μL pipette. Five imidacloprid concentrations and an acetone control were used. At least ten larvae were treated in each of the six groups and five control larvae were weighed prior to treatment. Daily observations for mortality were taken until pupation and at adult eclosion. Signs of larval intoxication (e.g., spasms, paralysis) and formation of malformed pupae were recorded. Dissections were performed on a subset of larvae that had AE. Photographs were taken with a Nikon DS-Ri2 digital camera connected to a Nikon SMZ1270 stereo microscope. The results obtained were compared to topical imidacloprid exposures conducted on fifth instar monarch butterfly^14,15^ to provide a more complete description of AE.

#### Imidacloprid toxicokinetics in AE-susceptible and unsusceptible species

Fifth instar monarch larvae and sixth instar fall armyworm larvae were topically treated with a nominal dose of 20 μg imidacloprid, as outlined in previous experiments. Five whole larval and pupal samples, each with a minimum mass of 400 mg for armyworms (two individuals) and 800 mg for monarchs (one individual) were collected at the start of an experiment and at the following time points post-treatment: 4 h (armyworms), 24 h (monarchs and armyworms), and after pupation or arrested ecdysis (monarchs and armyworms). All larval/pupal samples were stored individually in plastic containers at − 20 °C until quantification of imidacloprid and its metabolites.

All samples were analyzed for imidacloprid and its two metabolites, 5-hydroxy imidacloprid and imidacloprid olefin, which are toxic to insects^40^. Samples were individually homogenized using a mortar and pestle. A 0.2 g portion of the sample was extracted with acetonitrile. Approximately 1 mL of the extract was transferred to a dispersive solid phase extraction (dSPE) tube containing 150 mg MgSO4, 50 mg PSA, and 50 mg C18. Extracts were diluted with 50:50 methanol: water and internal standards (5-OH imidacloprid-d4, 13C, 15 N imidacloprid olefin, and imidacloprid-pyridine-d4-methylene-d2) were added prior to LC–MS analysis. An injection volume of 2 µL was used. A Vanquish Flex LC pump interfaced with a TSQ Altis triple quadrupole mass spectrometer (Thermo Fisher Scientific) were used for the analysis. All insecticides were analyzed in positive electrospray ionization mode. The MS ionization source conditions were as follows: spray voltage 3700 V, sheath gas 30 (Arb), auxiliary gas 6 (Arb), sweep gas 1 (Arb), ion transfer tube temperature 325 °C, and vaporizer temperature 350 °C. MS acquisition was performed in selected reaction monitoring (SRM) mode with argon used as the collision gas. Data analysis was performed using Xcalibur 4.2 software (Thermo Fisher Scientific).

Chromatographic separation was achieved using an AccucoreaQ column (100 × 2.1 mm, 2.6 µm). The column compartment temperature was 30 °C. Mobile Phase A was 2% methanol, 5 mM ammonium formate, and 0.1% formic acid in water and Mobile Phase B was 2% water, 5 mM ammonium formate, and 0.1% formic acid in methanol. The gradient conditions were as follows: start at 0% B, linear ramp to 100% B at 8 min, hold at 100% B for 2 min, drop to 0% B in half a min, and hold at 0% B for 1.5 min. The flow rate was 0.3 mL/min and the total run time of the method was 12 min. For both species, a calibration curve that ranged from 0.02 to 2.5 µg/g was prepared with control larvae. Quality control (QC) samples were prepared in triplicate at 0.3 µg/g and analyzed with experimental samples. All QC samples had a calculated concentration within 15% of the nominal value. The percent recovery was 99% for imidacloprid, 94% for 5-hydroxy imidacloprid, and 95% for imidacloprid olefin.

#### Effect of imidacloprid at various times prior to pupation

Sixth-instar corn earworm larvae were topically treated with 1 µL of 20 μg/µL imidacloprid-acetone solution at four different stages of molting as measured by stemmata pigment movement during head capsule slippage (HCS): (1) stemmata intact (several hours prior to HCS), (2) start of HCS, (3) 8 to 14 h after HCS (average 12 h), and (4) 20 to 24 h after HCS (average 23 h). A minimum of eight larvae were treated per stage. Acetone controls (n = 4 to 6) at each stage were employed. During the course of the experiments, signs of intoxication, daily mortality until pupation, pupal development, and adult eclosion were recorded. A subset of AE larvae was dissected and observed with the dissecting microscope.

Previously published papers suggested that the developmental window of susceptibility to AE varied between topical and dietary exposure routes^14,15^; this difference could be due to toxicokinetic factors. To evaluate differences in AE expression with the timing and route of exposure, 16 fifth instar monarch larvae were fed an average concentration of 0.5 μg of imidacloprid per g tropical milkweed leaf for 24 h. This concentration was obtained by applying 100 or 150 μL of a 0.01 μg/μL imidacloprid suspension on approximately 2 or 3 g of leaf material, respectively. Leaves were air-dried and photographs of leaves were taken prior to larval feeding and 24 h after feeding to obtain the surface area consumed by each larva, similar to methods described in^14^. Based on the estimated leaf concentration and surface area of leaf consumed, an oral imidacloprid dose consumed by each larva was estimated (the use of silwet ensured that one side of a leaf surface was entirely coated with the imidacloprid suspension). Five to six larval/pupal samples, with each sample corresponding to a minimum of 700 mg at the start of an experiment, were collected at various points (see Table S4). All larval/pupal samples were stored individually in plastic containers at − 20 °C until analysis for concentrations of imidacloprid and its two metabolites (see analytical methodology in “Imidacloprid toxicokinetics in AE-susceptible and unsusceptible species” section).

#### Observational details on pupation in corn earworms

Two-day old sixth instar corn earworms were treated with acetone and 20 µg imidacloprid (one µL of a 20 µg/µL solution) and observed continually over three days (except from 20:00 to 06:00) for initiation of apolysis, denoted by HCS as observed under a dissecting microscope, and the beginning of ecdysis, denoted by commencement of tracheal shedding at the posterior end. This information was obtained for 24 larvae treated with acetone or imidacloprid (n = 12 each). Time to landmark events were recorded.

### Statistical analyses

All statistical analyses were done in RStudio 1.4.1106 (R version 4.0.4)^41^. Data for all species were analyzed independently and figures were made using “ggplot2”^42^. To identify if there was a linear relationship between percent AE and imidacloprid doses or times of treatment, Pearson’s correlation test was performed and R (correlation coefficient) and *p* (significance) values were obtained. A poisson glm model and type 3 ANOVA (obtained from the “car” package^43^) were used to evaluate differences in days to pupation/AE between control and imidacloprid-treated larvae. Type 3 ANOVA was used to analyze differences in internal imidacloprid concentrations over time in monarch butterfly and fall armyworm larvae; if significance was obtained (*p* < 0.05), Dunnett’s test for multiple comparisons (“emmeans” package^44^) was employed to compare baseline concentrations (0-h) with concentrations measured at other time points. A Welch two sample t-test was used to analyze differences in the number of hours it took larvae in control and imidacloprid-treated groups to initiate pupal ecdysis following head capsule slippage.

## Supplementary Information

Supplementary Information.

## Data Availability

Data and metadata pertaining to this manuscript are publicly available at this GitHub repository: https://github.com/Niranjana296/Arrested-pupal-ecdysis.
