# Supplementary material for: Neonicotinoids can cause arrested pupal ecdysis in Lepidoptera
Source: Sci Rep. 2021 Aug 4;11:15787. doi: 10.1038/s41598-021-95284-0 (PMC8339065; doi:10.1038/s41598-021-95284-0)
Supplement: Supplementary file 1 — Supplementary Information. [file 41598_2021_95284_MOESM1_ESM.docx]

Figure S1. Arrested pupal ecdysis in representative final instar larvae of painted lady (top row), red admiral (middle row) and wax moth (bottom row) species. Careful removal of old larval cuticle from painted ladies and red admirals and an examination of wax moths showed complete pupal case on the dorsal and posterior side and unshed tracheal lining. In all three species the appendages had not expanded and the ventral side of the first abdominal segments were not sclerotized.


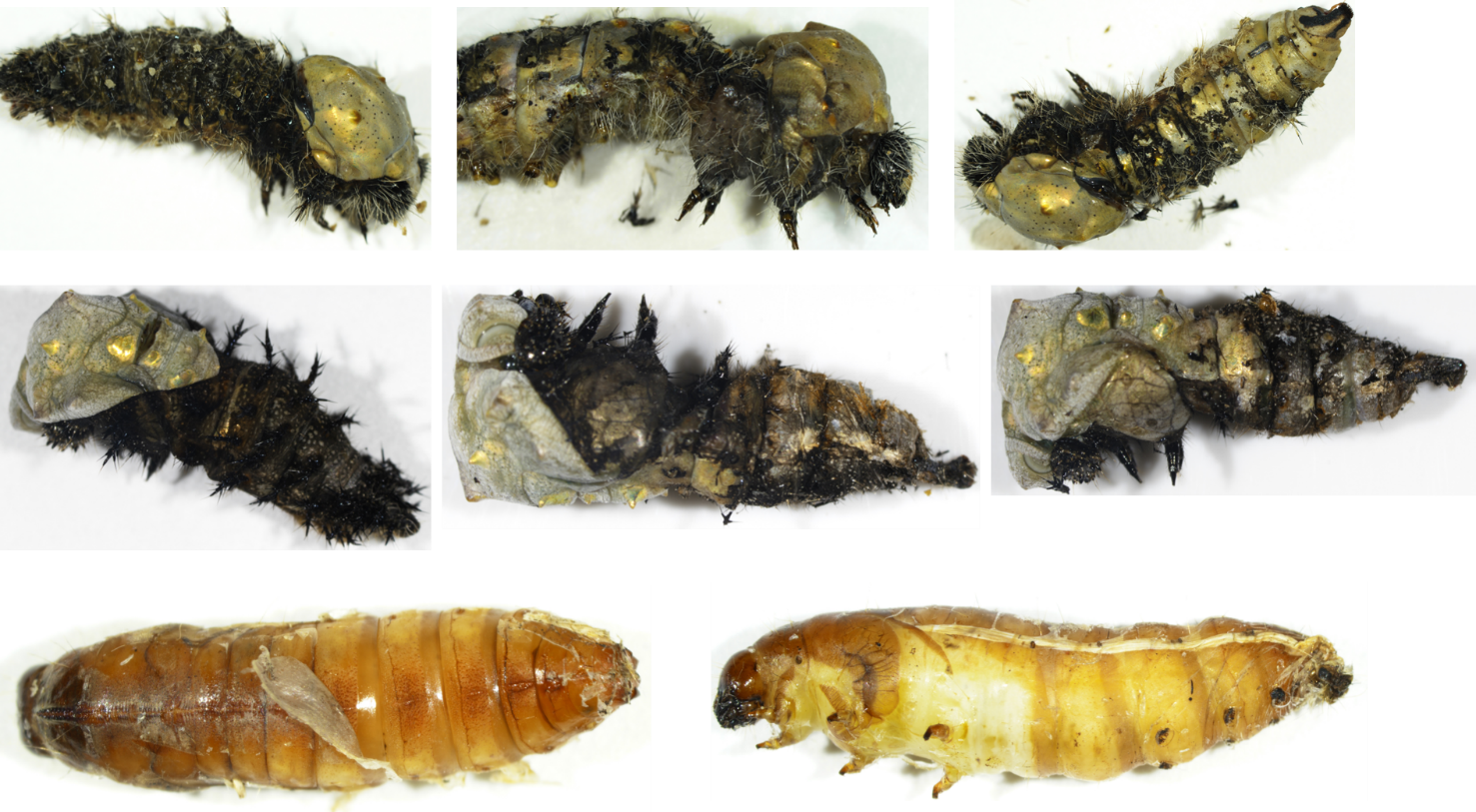


Figure S2. Representative corn earworms that had pupated following topical treatment with 20 µg imidacloprid. A. Treatment occurred 12 hours after head capsule slippage. Pupa had wrinkled appendages, some blood loss, and bloated wings. B. Treatment occurred 23 hours after head capsule slippage resulting in a normal looking pupa. Removal of pupal cuticle ca. 2 weeks later showed a completely developed adult and mostly expanded appendages, except for deformed/uninflated wings (right panels). All panels showing the ventral side.


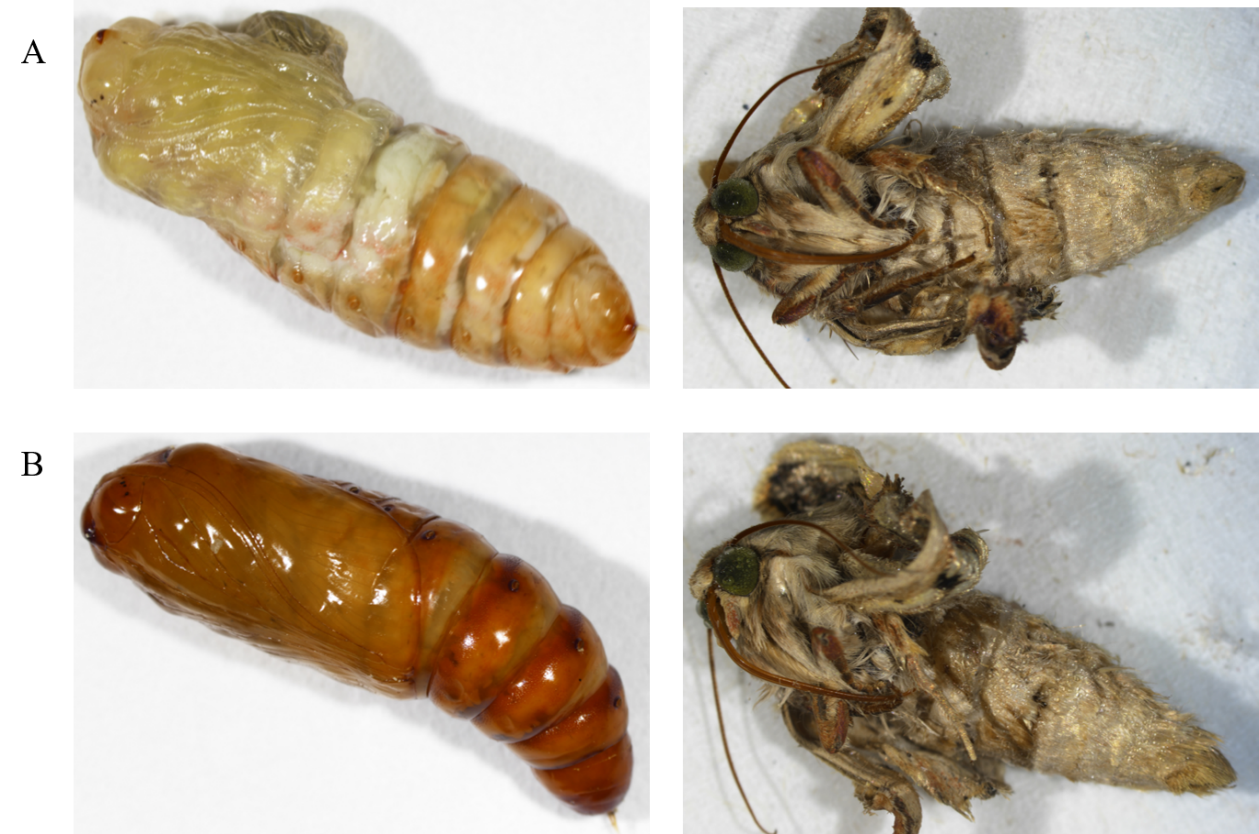


Figure S3. Careful removal of unshed larval cuticle from corn earworm sixth instars that were topically treated with 20 µg imidacloprid and that had just exhibited arrested ecdysis showed a completely developed pupal case throughout the body. Abdominal segments were starting to tan. Appendages were unexpanded and untanned. Arrow points to antennae.


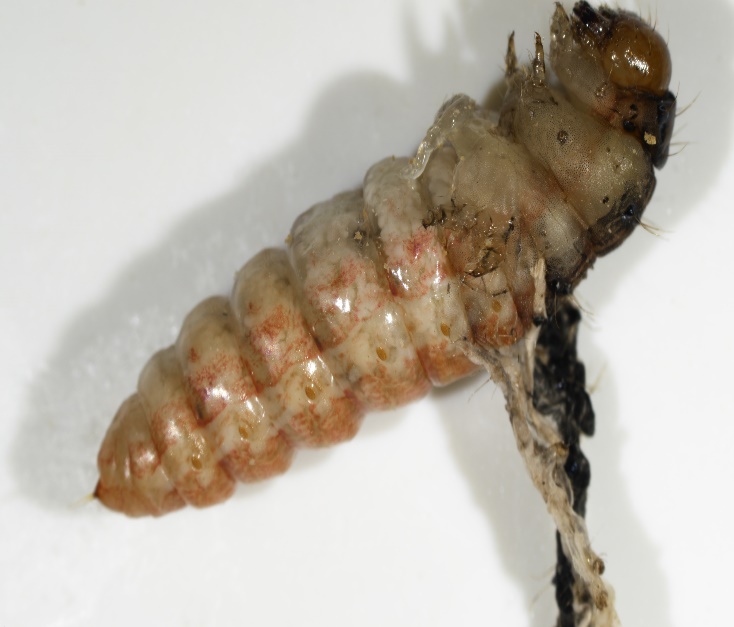

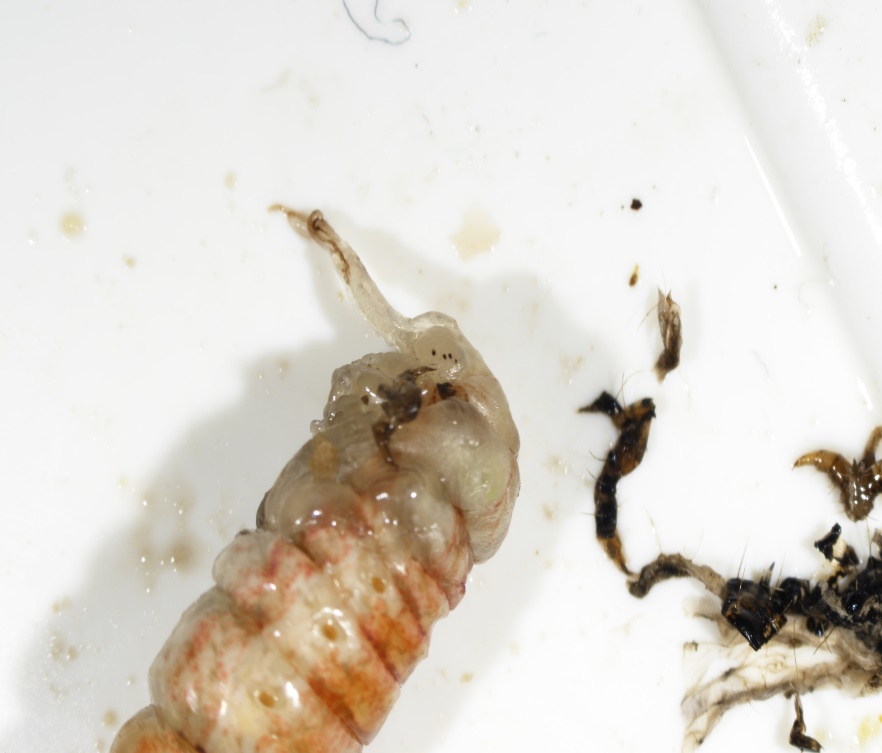


Figure S4. Stacked barplot depicting the percent of imidacloprid-treated fifth instar (penultimate instar) corn earworms that had larval mortality, arrested pupal ecdysis (AE), pupation with no adult eclosion, and pupation with adult eclosion. Larvae were topically treated with six doses including a control (n = 10 larvae/dose).


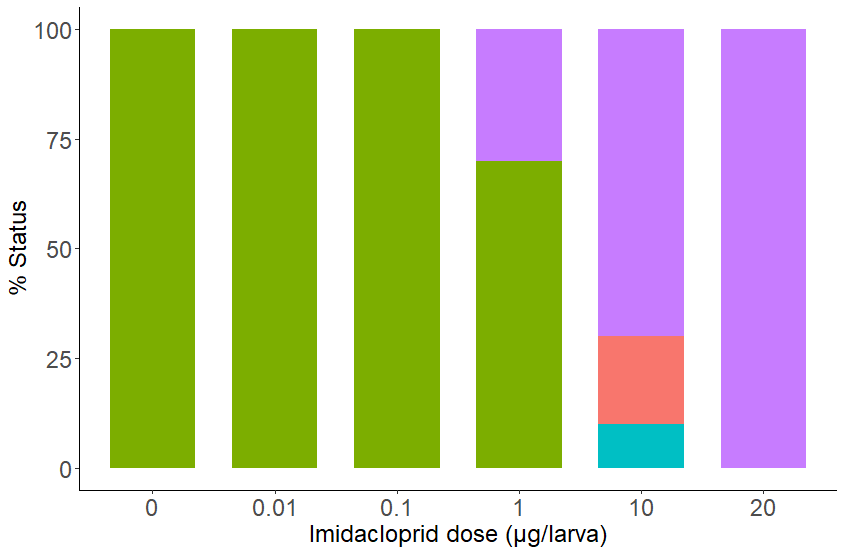


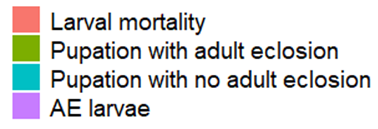


Table S1. Effect of topical application of 60 and 100µg imidacloprid on final instar monarch butterfly, European corn borer, and fall armyworm larvae

| Species | Dose (µg/larva)^a^ | Percent status | | | | Mean (± SD) days to pupal ecdysis^c^ |
| --- | --- | --- | --- | --- | --- | --- |
|  |  | Larval mortality^b^ | AE | Adult eclosion | No eclosion |  |
| Monarch butterfly | 60 | 70 | 30 | 0 | 0 | 5 (± 1) |
|  | 100 | 100 | 0 | 0 | 0 | NA |
| European corn borer | 100 | 50 | 0 | 10 | 40 | 6.8 (± 1.1) |
| Fall armyworm | 60 | 0 | 0 | 100 | 0 | 3.2 (± 0.9) |
|  | 100 | 0 | 0 | 100 | 0 | 3.2 (± 1.0) |

^a^ Three and five µL of 20 µg/µL imidacloprid-acetone dose was applied on the dorsal prothorax of each larva to obtain larval doses of 60 and 100 µg imidacloprid, respectively; 10 larvae were treated per dose per species.

^b^ Mortality prior to pupation and with symptoms of intoxication.

^c^ Includes both days to successful pupation and AE.

AE: arrested pupal ecdysis; SD: standard deviation

Table S2. Mean days to pupal ecdysis in final-instar larvae of seven species topically treated with five imidacloprid doses and acetone (control)

| Species  (weight)^a^ | Dose (μg/  larva) | # initiated pupal ecdysis^b^ (total treated) | Mean (± SD) days to pupal ecdysis | Statistical analyses^c^ |
| --- | --- | --- | --- | --- |
| Monarch butterfly^d^  (1.1 ± 0.25 g) | 0 | 32 (32) | 3.3 (± 1.4) | χ^2^ = 2.83; df = 5; p = 0.727 |
|  | 0.01 | 22 (22) | 2.8 (± 1.1) |  |
|  | 0.1 | 33 (33) | 3.5 (± 1.1) |  |
|  | 1.0 | 33 (33) | 3.2 (± 1.0) |  |
|  | 10 | 22 (22) | 3.0 (± 0.6) |  |
|  | 20 | 10 (10) | 2.7 (± 0.8) |  |
| Painted lady  (0.66 ± 0.18 g) | 0 | 10 (10) | 3.4 (± 1.0) | χ^2^ = 1.80; df = 5; p = 0.877 |
|  | 0.01 | 10 (10) | 3.8 (± 0.6) |  |
|  | 0.1 | 10 (10) | 3.4 (± 1.0) |  |
|  | 1.0 | 10 (10) | 3.9 (± 1.3) |  |
|  | 10 | 10 (10) | 3.8 (± 1.1) |  |
|  | 20 | 10 (10) | 4.4 (± 0.7) |  |
| Red admiral  (0.15 ± 0.04 g) | 0 | 10 (10) | 4.9 (± 0.7) | χ^2^ = 6.07; df = 5; p = 0.299 |
|  | 0.01 | 9 (9) | 3.7 (± 0.9) |  |
|  | 0.1 | 9 (9) | 3.4 (± 1.0) |  |
|  | 1.0 | 9 (10) | 3.0 (± 1.7) |  |
|  | 10 | 10 (10) | 3.7 (± 1.4) |  |
|  | 20 | 9 (10) | 4.6 (± 1.1) |  |
| Corn earworm  (0.49 ± 0.11 g) | 0 | 10 (10) | 4.4 (± 0.7) | χ^2^ = 0.63; df = 5; p = 0.987 |
|  | 0.01 | 10 (10) | 4.5 (± 0.5) |  |
|  | 0.1 | 10 (10) | 4.8 (± 0.4) |  |
|  | 1.0 | 10 (10) | 4.3 (± 0.5) |  |
|  | 10 | 10 (10) | 4.3 (± 0.5) |  |
|  | 20 | 10 (10) | 4.1 (± 0.3) |  |
| Wax moth  (0.24 ± 0.06 g) | 0 | 15 (15) | 4.3 (± 1.3) | χ^2^ = 2.86; df = 5; p = 0.722 |
|  | 0.01 | 15 (15) | 4.1 (± 1.1) |  |
|  | 0.1 | 15 (15) | 4.2 (± 1.1) |  |
|  | 1.0 | 16 (16) | 4.3 (± 0.7) |  |
|  | 10 | 15 (15) | 4.7 (± 1.1) |  |
|  | 20 | 9 (16) | 5.4 (± 0.5) |  |
| European corn borer  (0.06 ± 0.02 g) | 0 | 15 (15) | 5.1 (± 2.0) | χ^2^ = 6.32; df = 5; p = 0.277 |
|  | 0.01 | 15 (15) | 5.3 (± 1.7) |  |
|  | 0.1 | 13 (14) | 5.9 (± 1.9) |  |
|  | 1.0 | 15 (15) | 5.9 (± 1.7) |  |
|  | 10 | 12 (15) | 6.0 (± 1.9) |  |
|  | 20 | 11 (15) | 7.4 (± 0.9) |  |
| Fall armyworm  (0.37 ± 0.07 g) | 0 | 10 (10) | 3.9 (± 0.6) | χ^2^ = 1.64; df = 5; p = 0.897 |
|  | 0.01 | 9 (10) | 3.7 (± 0.7) |  |
|  | 0.1 | 10 (10) | 3.2 (± 0.8) |  |
|  | 1.0 | 10 (10) | 3.2 (± 0.4) |  |
|  | 10 | 10 (10) | 3.1 (± 0.3) |  |
|  | 20 | 10 (10) | 3.1 (± 0.3) |  |

^a^ Based on the weight (in grams) of five to ten control larvae at the time of treatment. For monarchs, weights based on treating 18 control larvae.

^b^ Included both larvae that pupated and larvae that underwent AE.

^c^ A poisson glm model and ANOVA were used to analyze differences in number of days to initiation of pupal ecdysis between controls and treatments for each species.

^d^ Monarch butterfly data were obtained from Krishnan et al. (2020).

#: number of larvae that initiated pupal ecdysis; SD: standard deviation

Table S3. Internal concentrations of imidacloprid and its metabolites in final instar larvae of fall armyworm and monarch butterfly following topical exposure^a^

| Species | Collection endpoint/time following treatment | # of samples analyzed^b^ | Sample mean (± SD) weight at time of treatment (g) | Mean (± SD) measured internal dose (µg/g insect)^c^ |
| --- | --- | --- | --- | --- |
| Fall armyworm | 0 h | 4^d^ | 0.65 (± 0.07) | 59 (± 11) parent IMI (n = 4)  0.4 (± 0.05) 5-hydroxy IMI (n = 2) |
|  | 4 h | 5 | 0.55 (± 0.07) | 56 (± 23) parent IMI (n = 5)  0.6 (± 0.2) 5-hydroxy IMI (n = 3)  2.1 (± 0.9) IMI olefin (n = 2) |
|  | 24 h | 5 | 0.54 (± 0.12) | 13 (± 7.9) parent IMI (n = 5)  0.3 (± 0.2) 5-hydroxy IMI (n = 2)  1.4 (± 0.4) IMI olefin (n = 3) |
|  | Pupa (~ 96 h) | 5 | 0.55 (± 0.15) | < 0.02 parent IMI (n = 2) |
| Monarch  butterfly | 0 h | 5 | 1.1 (± 0.09) | 24 (± 10) parent IMI (n = 5) |
|  | 24 h | 5 | 0.87 (± 0.07) | 31 (± 12) parent IMI (n = 5) |
|  | AE (~ 48 h) | 5 | 1.0 (± 0.04) | 20 (± 3.4) parent IMI (n = 5) |

^a^ One µL of 20 µg/µL imidacloprid-acetone dose was applied on the dorsal prothorax of each larva

^b^ A fall armyworm sample consisted of two larvae and a monarch butterfly sample consisted of a single larva.

^c^ Imidacloprid parent and metabolite doses in larval/pupal samples were measured through LC/MS-MS. Limit of quantification was 0.02 µg/g.

^d^ A sample was excluded from analyses (3X greater concentration provided).

#: number of samples analyzed; SD: standard deviation; AE: arrested pupal ecdysis; n: number of samples with detectable doses.

Table S4. Dietary imidacloprid exposure on final instar monarch butterfly: larval effects and internal concentrations

| Treatment^a^ | # of larvae treated | Mean (± SD) weight at time of treatment (g) | Time to pupal ecdysis^b^ | Mean dose consumed^c^  (µg) | Effect | Mean (± SD) measured internal dose (µg/g monarch)^d^ |
| --- | --- | --- | --- | --- | --- | --- |
| 24 h feeding on treated leaves | 5 | 0.99 (± 0.08) | 38 h  (1 to 2 days) | 1.2 (± 0.40) | 100% AE | 0.14 (± 0.06) parent IMI (n = 5) |
| 24 h feeding on treated leaves + 24 h feeding on untreated leaves | 6 | 0.74 (± 0.03) | 60 to 72 h  (2 to 3 days) | 0.86 (± 0.23) | 100% pupae | < 0.02 parent IMI  (n = 6) |

^a^ Each larva was provided 0.5 µg of imidacloprid/g tropical milkweed leaf for 24 hours.

^b^ Approximate time to initiation of pupal ecdysis from start of treatment.

^c^ Calculated through methods described in Krishnan et al. (2021).

^d^ Imidacloprid parent and metabolite doses in larval/pupal samples were measured through LC/MS-MS. Limit of quantification was 0.02 µg/g. Additionally, 5 larvae provided the same imidacloprid leaf concentrations (and 0.68 ± 0.48 µg dose) for 24 hours were analyzed immediately thereafter, prior to initiation of pupal ecdysis, and found to contain 0.11 (± 0.04) µg/g parent imidacloprid.

#: number of samples analyzed; SD: standard deviation; AE: arrested pupal ecdysis; n: number of samples with detectable doses.
